# Cervical Funneling: Potential Pitfall of Point-of-Care Pelvic Ultrasound

**DOI:** 10.7759/cureus.1649

**Published:** 2017-09-03

**Authors:** Lori A Stolz, Richard Amini, Elaine H Situ-LaCasse, Faryal Shareef, Heather A Reed, Srikar Adhikari

**Affiliations:** 1 Department of Emergency Medicine, University of Cincinnati; 2 Department of Emergency Medicine, University of Arizona; 3 College of Medicine, University of Arizona; 4 Department of Obstetrics and Gynecology, University of Arizona

**Keywords:** cervical length, cervical funneling, point of care ultrasound, bedside ultrasound, pregnancy

## Abstract

Though point-of-care ultrasound applications continue to expand, there are findings that are not within the scope of emergency ultrasound. It is important for emergency physicians to be aware of incidental findings that can be identified on comprehensive ultrasounds performed by other imaging departments in order to fully understand the limitations of bedside ultrasound. In this case, a gravid patient presented to the emergency department with pelvic cramping and vaginal bleeding. Point-of-care transabdominal pelvic ultrasound examination was performed and demonstrated cervical funneling. In the appropriate patient, cervical insufficiency due to cervical funneling may be an indication for cerclage in a pregnant patient.

## Introduction

Point-of-care pelvic ultrasound is a key component in the evaluation of pregnant patients presenting to the emergency department (ED) with complaints of abdominal pain, cramping or vaginal bleeding. The usual management of a pregnant patient prior to fetal viability (generally less than 24 weeks) is focused on screening for IUP and evaluating for maternal emergency. The American College of Emergency Physicians endorses the use of bedside ultrasound for detection of intrauterine pregnancy, ectopic pregnancy, fetal heart rate in all stages of pregnancy, dating of the pregnancy, and detection of significant free fluid [1]. Comprehensive radiology-performed ultrasound examinations include evaluation of additional elements that can impact patient care. This case highlights a rare case of a pre-viability fetal finding that can impact management.

## Case presentation

**Figure 1:**
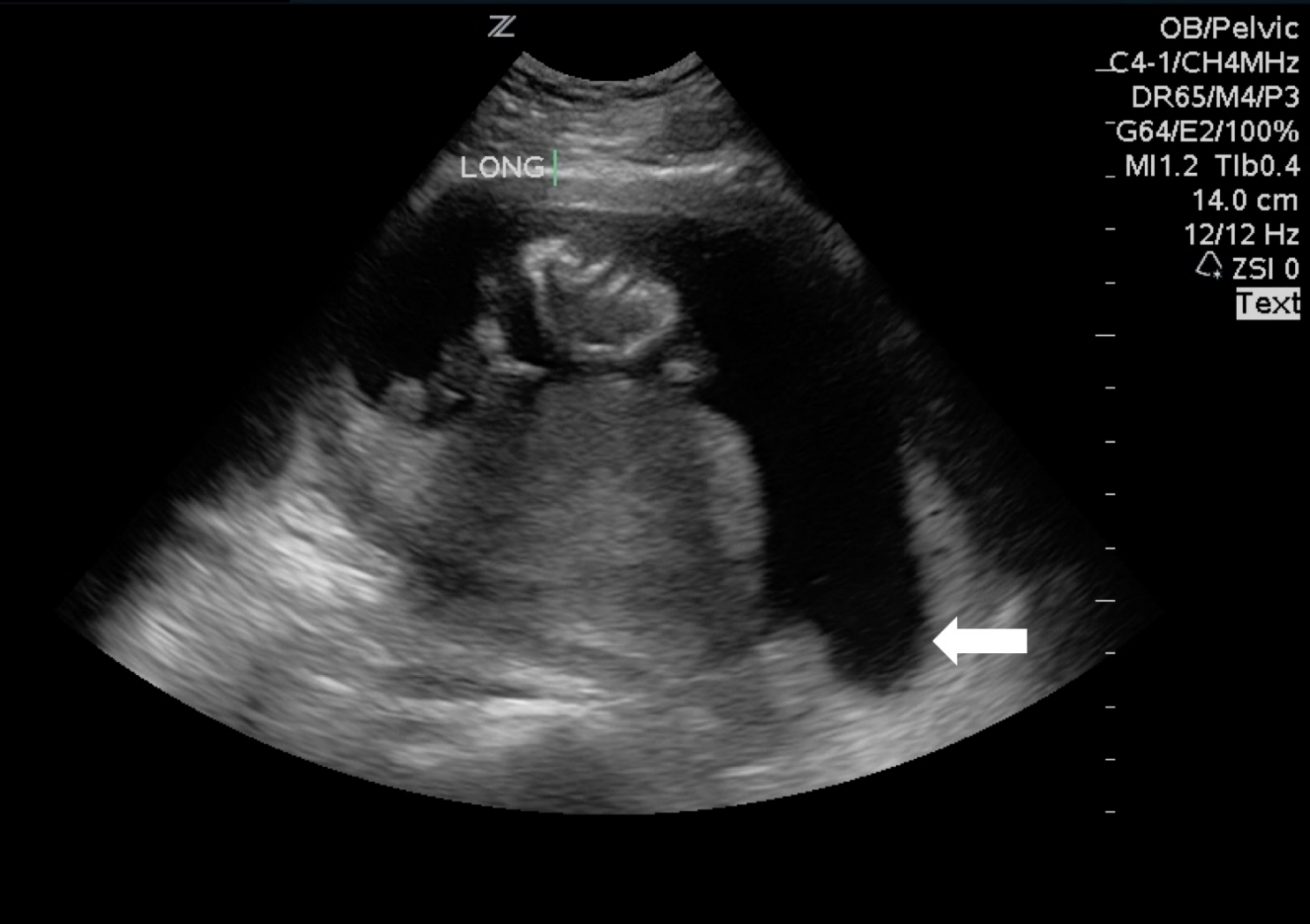
Sagittal imaging of a gravid uterus. Point of care sonographic image of uterus with cervical funneling (white arrow).

A 27-year-old female G2P1 at 16 weeks and six days gestation by last menstrual period (LMP) presented to the ED to be evaluated for pelvic pressure and cramping with light vaginal bleeding. The patient denied any loss of amniotic fluid and reported one previous uncomplicated pregnancy. Examination revealed vital signs within normal limits, blood present at the vaginal vault, closed os and otherwise unremarkable examination. Point-of-care transabdominal ultrasound examination was performed and demonstrated a live intrauterine pregnancy with a fetal heart rate of 160 beats per minute and gestational age measurements which were consistent with dates by LMP. The patient was discharged home with recommendations to follow up with her obstetrician. She presented two days later and was found to have cervical os dilated to 3 cm, oligohydramnios and fetal demise. Upon review of the initial point-of-care ultrasound performed, cervical funneling was present (Figure 1).

## Discussion

**Figure 2:**
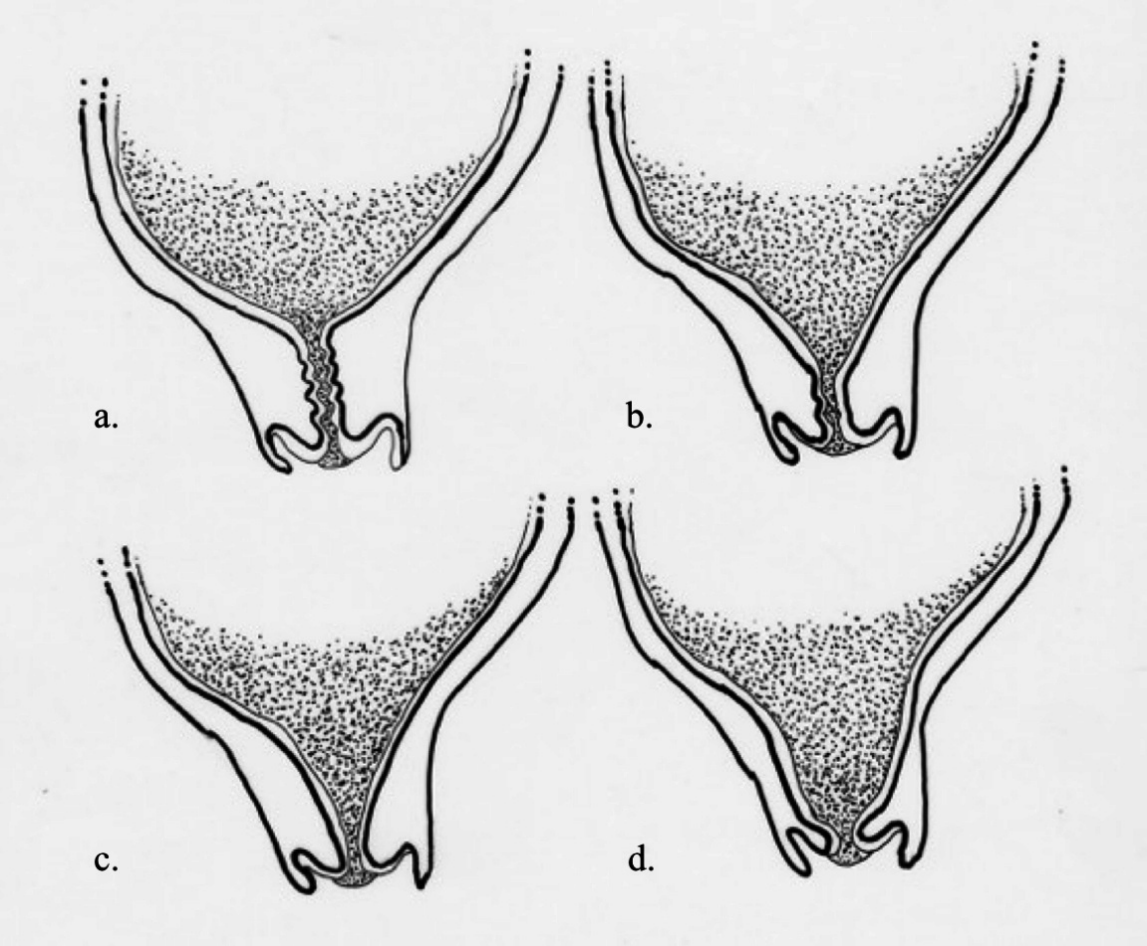
Cervical progression in pregnancy. Progressive stages of cervical dilatation: T-shape (a), Y-shape (b), V-shape (c), U-shape (d).

Pregnant women found to have cervical funneling are at increased risk of spontaneous preterm delivery (usually occurring in the second trimester) [2]. The sonographic findings of cervical funneling are also associated with chorioamnionitis, abruption, rupture of the membranes and neonatal morbidity and mortality [3]. Cervical funneling is defined sonographically as a protrusion of amniotic membranes into the internal cervical os by greater than 5 mm from the shoulder of the original internal os as measured along the lateral border of the funnel [4]. This finding is usually accompanied by short cervical length (defined as <25 mm). The shape of the funnel indicates the degree of cervical dilation and effacement. With cervical ripening, the cervix progresses from a T-shape (with no funnel) to a Y-shape (with amniotic membrane protrusion into the internal os with a closed external), to a V-shape (with funneling of the amniotic membranes nearly to the external os) and finally a U-shape (Figure 2) [5]. U- or V-shaped funnels are a more ominous finding, indicating more advanced cervical ripening. Although cervical funneling can be diagnosed transabdominally, it is best evaluated and diagnosed via transvaginal sonography with the cervix in the sagittal plane. In this plane, the internal and external os are visualized simultaneously. Fundal pressure is applied and the sonographer notes the presence or absence of amniotic membrane prolapse into the cervical canal. Prolapse of the membranes more than 5 mm is a positive exam and the shape of the funnel should be noted.

Cervical funneling is a marker of cervical insufficiency, which is characterized by painless cervical dilation after the first trimester and associated miscarriage or preterm delivery. The risks associated with cervical funneling may be mitigated by placing an emergency cerclage in which a stitch is placed in the cervix. Cervical insufficiency is a difficult diagnosis to make and often requires a history of prior pregnancy loss related to painless cervical dilation or spontaneous preterm birth, however ultrasonographic findings may aid in the diagnosis [6].

Ultrasonographic findings of cervical funneling combined with a history of preterm birth (resulting from cervical insufficiency) are criteria for the placement a cervical cerclage in a patient with a singleton pregnancy between 16 and 24 weeks of gestation [6]. In this case, the patient had a previously uncomplicated pregnancy and thus would not have met criteria for cerclage. However, cerclage in the appropriate patient may be beneficial in reducing preterm birth and neonatal morbidity and mortality. In patients without a history of preterm birth, studies have not shown a reduction in preterm birth when cerclage is placed based on sonographic findings alone [6].

Evaluation of the cervix is not considered a standard element of point-of-care pelvic ultrasound evaluation (transabdominal or transvaginal). In this case, the length of the cervix was not measured, however the findings of cervical funneling were present on the point-of-care transabdominal ultrasound examination but were not recognized. Additionally, clinicians should be aware that transabdominal cervical length measurements may overestimate the cervical length. Therefore, when concern for cervical funneling is present, comprehensive (imaging department) transvaginal ultrasonography is recommended for definitive diagnosis [7].

## Conclusions

Identification of cervical funneling on point-of-care ultrasound (transabdominal or transvaginal) can help with timely consultation and appropriate antepartum interventions when necessary. Thus, clinicians who perform point-of-care pelvic ultrasonography should be aware of the sonographic findings found with cervical funneling and its associated risk for preterm labor.
